# Efficacy of an Acid-Oxidizing Solution against Mycobacterium ulcerans

**DOI:** 10.1128/AAC.00870-21

**Published:** 2022-01-18

**Authors:** Louisa Warryn, Gerd Pluschke

**Affiliations:** a Swiss Tropical and Public Health Institute, Basel, Switzerland; b University of Basel, Basel, Switzerland

**Keywords:** Buruli ulcer, *Mycobacterium ulcerans*, acid-oxidizing solution, wound management

## Abstract

For the treatment of chronic wounds, acid-oxidizing solutions (AOSs) with broad-spectrum microbicidal activity without disturbing granulation tissue formation have been developed. We found AOSs to efficiently kill Mycobacterium ulcerans, the causative agent of Buruli ulcer, which is able to survive harsh decontamination treatments. Topical AOS treatment of Buruli ulcer lesions may support the recommended antibiotic therapy (oral rifampin and clarithromycin), prevent contamination of the environment by the mycobacteria, and control secondary infections, which are a prevalent wound management problem in resource-poor settings where Buruli ulcer is endemic.

## INTRODUCTION

Mycobacterium ulcerans disease—or Buruli ulcer (BU)—is a chronic necrotizing infectious disease afflicting skin and soft tissue (1). The pathology of this disease, affecting primarily children in West and Central Africa, is largely attributed to the production of the polyketide cytotoxin mycolactone by M. ulcerans (2). Although BU is treatable by an 8-week regimen of daily rifampin and clarithromycin (3), the destruction to skin and soft tissue may necessitate adjunctive surgical intervention for complete resolution. Secondary colonization of BU lesions by other bacteria, including Staphylococcus aureus and Pseudomonas aeruginosa, is common (4) and may delay wound healing and cause other complications.

Chronic wounds do not progress through the typical phases of healing, and as BU lesions have features of chronic wounds, approaches developed for managing chronic wounds of other etiologies may be applicable to BU treatment. Chronic wounds tend to have an alkaline pH and a bioburden in the form of biofilms, and studies have shown that a reduction of pH to acidic levels supports healing (5). Consequently, efforts have been made to develop new wound care regimens that acidify chronic wounds to facilitate healing.

An acid-oxidizing solution (AOS, Applied Pharma Research, Balerna, Switzerland) has been developed for the treatment of chronic wounds. This AOS formulation is based on hypochlorous acid (which represents >95% of the total free chlorine species in the solution) with a low pH (2.5–3.0) and high reduction-oxidation (redox) potential. As such, it has a three-pronged approach to promoting wound healing: (i) hypochlorous acid, which is broadly microbicidal (6), (ii) low pH that is refractory to microbial growth in wounds (7), and (iii) high redox potential, which destabilizes the membrane potential of microorganisms and facilitates their killing (8). The AOS was found to have broad-spectrum microbicidal activity, and was neither toxic nor sensitizing to skin, mucosal membranes, or eyes. (9–11).

Improving BU healing by way of wound acidification has been explored previously. Acidified nitrate was shown to be bactericidal to M. ulcerans *in vitro* (12) and to aid wound size reduction in BU lesions. Neither acidic pH alone nor nitrite alone was found to lead to bacterial killing (13). Therefore, here we assessed *in vitro* killing of M. ulcerans following exposure to AOS.

Two different formulations of AOS, produced with a patented Tehclo Technology, were tested: AOS formulation 1 contains 40–70 mg/L of stabilized hypochlorous acid with a redox potential between 1000 and 1200 mV, while AOS formulation 2 contains 70–100 mg/L of stabilized hypochlorous acid with a redox potential between 1000 and 1300 mV. Both AOS solutions have low pH (2.5–3.0). AOS formulation 1 is approved as medical device class III with ancillary antimicrobial action in Europe and under 510k regulation in the US with the indication of debridement, irrigation, cleansing, and moistening of acute and chronic wounds (e.g., diabetic foot ulcers, pressure ulcers, vascular ulcers).

The M. ulcerans strain S1013, a low-passage Cameroonian clinical isolate (14), was grown for 8 weeks in Middlebrook 7H9 medium (Becton-Dickinson), supplemented with 0.2% glycerol (Sigma) and 10% OADC (oleic acid, albumin, dextrose, catalase; Becton-Dickinson), before being used in the tests. Cultures (approximately 10^6^ CFU/mL) were exposed to the test solutions in a 1:20 ratio (50 μL of culture to 950 μL of AOS) for varying lengths of time, after which the suspension was centrifuged at 13,300 × *g* for 1 min to pellet the bacteria. The supernatant was immediately removed and the bacterial pellet resuspended in 200 μL of Middlebrook 7H9 liquid medium supplemented with 0.2% glycerol and 10% OADC. For resazurin tests, 20 μL of a resazurin solution (0.125 mg/mL; Sigma) was added to the treated cells, the culture was incubated at 30°C for 3 days after which the fluorescence was measured, and the metabolic activity was calculated relative to the included controls. Alternatively, the treated cells were plated out on Middlebrook 7H9 agar medium supplemented with 0.2% glycerol and 10% OADC, and incubated at 30°C for up to 6 months. CFU were counted monthly, and the final count was done at the end of the experiment.

Efficacy of AOS formulation 1 was further assessed by adding 950 μL of AOS to 50 μL of M. ulcerans cultures containing different amounts of human serum, from no serum up to 50% serum. Additionally, M. ulcerans cultures of 5 × 10^5^ – 4 × 10^6^ CFU/mL were tested to see the efficacy of AOS formulation 1 against particularly heavy doses of the bacteria. Finally, tests were done to see how efficacious AOS 1 was when used in lower excesses relative to the bacterial inoculum. For this, M. ulcerans cultures (10^6^ CFU/mL) were exposed to AOS 1 in ratios of 1:20 (50 μL bacteria culture and 950 μL AOS 1), 1:10 (50 μL bacteria culture, 50 μL culture medium, and 900 μL AOS 1), 1:5 (50 μL bacteria culture, 150 μL culture medium, and 800 μL AOS 1), and 1:2 (50 μL bacteria culture, 450 μL culture medium, and 500 μL AOS 1). The resazurin assay was used for all these additional tests, and the bacteria were exposed to the AOS test solution for a total of 10 min prior to plating.

**FIG 1:**
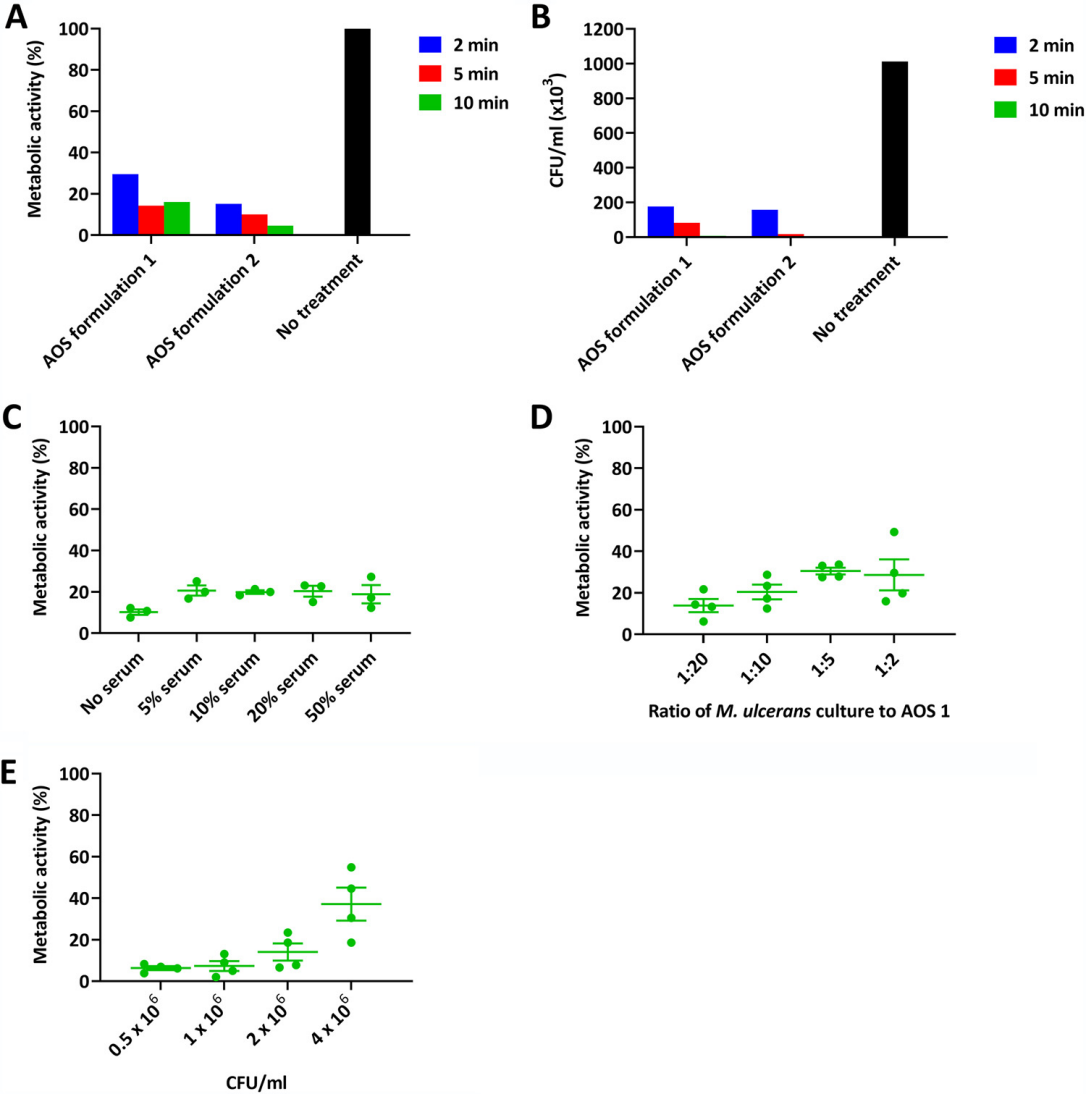
(A) Reduction in M. ulcerans metabolic activity as measured by the resazurin assay following exposure to AOS formulations 1 and 2 for 2, 5, or 10 min; untreated bacteria were included as controls. (B) Reduction in M. ulcerans CFU following exposure to AOS formulations 1 and 2 for 2, 5, or 10 min; untreated bacteria were included as controls. (C) Metabolic activity of bacterial suspensions containing different concentrations of human serum following a 10-minute exposure to AOS formulation 1; triplicate results are shown with the plotted means. (D) Metabolic activity of bacterial suspensions exposed to varying excesses of AOS formulation 1; quadruplicate results are shown with the plotted means. (E) Metabolic activity of bacterial suspensions containing increasing amounts of M. ulcerans following a 10-minute exposure to AOS formulation 1; quadruplicate results are shown with the plotted means. For the experimental results shown in panels A, B, C, and E, bacterial suspensions tested contained 10^6^ CFU/mL.

Initial resazurin tests revealed a time-dependent reduction in M. ulcerans metabolic activity upon exposure to both AOS formulations (Fig. 1A). A 2-min exposure to AOS formulations 1 and 2 resulted in a 70.5% and 84.3% reduction, respectively. Increasing the exposure time resulted in over 85% reduction with AOS formulation 1, and over 95% reduction with AOS formulation 2. CFU count-based analyses revealed a similar picture with an 82.6% and 84.5% reduction in CFU following a 2-min exposure to AOS formulations 1 and 2, respectively, relative to unexposed bacteria, and >99% reduction following 10-min exposure to both formulations (Fig. 1B). Formulation 1, which is approved both in the EU and the USA for chronic wound management, was selected for further tests.

As BU lesions have varying amounts of serum-rich exudate, we assessed the efficacy of the AOS in the presence of human serum. This resulted in slight reduction of AOS formulation 1 efficacy, but there was still over 75% reduction in metabolic activity even in bacterial suspensions containing 50% human serum (Fig. 1C). Interestingly, the efficacy of the AOS formulation did not decline much with increasing serum concentrations.

The 1-in-20 mixture of bacterial inoculum and AOS were chosen to reflect the recommended clinical application of AOS, which involves application to wetness. To ascertain that lower excesses of the AOS were still efficacious, we exposed M. ulcerans cultures to AOS formulation 1 in 1-in-20, 1-in-10, 1-in-5, and 1-in-2 ratios. While the standard 1-in-20 ratio was the most efficacious with an 86.1% reduction in metabolic activity, lower excesses of the AOS formulation could reduce M. ulcerans metabolic activity by >70% (Fig. 1D).

While the inoculum dose of 10^6^ CFU/mL selected for the previous analyses is well above the range routinely used for drug screens (14), we assessed elimination of even larger doses by the AOS formulation. As expected, there was some dose-dependent reduction in AOS efficacy with increasing inoculum sizes (Fig. 1E). Nevertheless, a >90% reduction in metabolic activity was achievable even with a starting inoculum of 2 × 10^6^ CFU/mL. At the highest inoculum dose tested (4 × 10^6^ CFU/mL), metabolic activity was still reduced by 62.83%.

Effective wound management is necessary in addition to specific chemotherapy for healing of BU lesions. These could involve simple cleansing and dressing, debridement, skin grafting, and prevention of secondary infections (15). AOS treatment may complement antibiotic treatment by directly killing M. ulcerans, and improving wound healing by eliminating secondary infections and stimulating a favorable wound microenvironment that fosters healing.

Clinical reports have demonstrated the efficacy of AOS formulation 1 when incorporated into the management of chronic wounds, with improved clinical outcomes following both inpatient and outpatient treatment (9–11). Low pH, hypochlorous acid, and high redox potential account for the broad-spectrum activity of AOS against microorganisms, including M. ulcerans, which is able to survive harsh decontamination procedures (16). In addition, the observed clinical efficacy of AOS formulation 1 could be partly explained by prevention of biofilm formation and the deterioration of already formed biofilms (17). Since M. ulcerans also adopts biofilm-like structures (18), the anti-biofilm activity of the AOS could also aid in the clearance of M. ulcerans from BU lesions.

While direct human-to-human transmission of M. ulcerans seems to be very rare, chronic human BU lesions may contribute to transmission by seeding environmental reservoirs. AOS treatment could reduce the spread of the pathogen into the environment. The simplicity of use of the AOS (thanks to the spray formulation) could allow patients to be treated at home, either by self-administration or by a health worker, thus reducing the need for repeated hospital visits for those patients in remote areas who reside far away from a primary health post. Clinical studies are needed to assess whether AOS treatment is a suitable adjunct to the WHO recommended BU treatment, and evaluate how translatable these results are into routine BU treatment.
